# Analgesic and anti‐inflammatory properties of novel, selective, and potent EP4 receptor antagonists

**DOI:** 10.1002/prp2.316

**Published:** 2017-05-14

**Authors:** Srinivasan Chandrasekhar, Xiao‐Peng Yu, Anita K. Harvey, Jennifer L. Oskins, Chaohua Lin, Xushan Wang, Maria‐Jesus Blanco, Matthew J. Fisher, Steven L. Kuklish, Matthew A. Schiffler, Tatiana Vetman, Alan M. Warshawsky, Jeremy S. York, Alison M. Bendele, Mark G. Chambers

**Affiliations:** ^1^ Lilly Research LaboratoriesEli Lilly and CompanyIndianapolisIndiana46285; ^2^ Bolder BioPATH Inc.BoulderColorado80309

**Keywords:** Anti‐inflammatory drugs, arthritis, EP4 antagonist, inflammation, pain, prostaglandin

## Abstract

Prostaglandin (PG) E_2_ is the key driver of inflammation associated with arthritic conditions. Inhibitors of PGE_2_ production (NSAIDs and Coxibs) are used to treat these conditions, but carry significant side effect risks due to the inhibition of all prostanoids that play important physiological function. The activities of PGE_2_ are transduced through various receptor sub‐types. Prostaglandin E_2_ type 4 receptor (EP4) is associated with the development of inflammation and autoimmunity. We therefore are interested in identifying novel EP4 antagonists to treat the signs and symptoms of arthritis without the potential side effects of PGE_2_ modulators such as NSAIDs and Coxibs. Novel EP4 antagonists representing distinct chemical scaffolds were identified using a variety of in vitro functional assays and were shown to be selective and potent. The compounds were shown to be efficacious in animal models of analgesia, inflammation, and arthritis.

**Abbreviation**
AIA: adjuvant induced arthritis
CIA: collagen induced arthritis
coxibs: cyclooxygenase inhibitors
cPGES: cytosolic prostaglandin E synthase
EP1, EP2, EP3, EP4: prostaglandin E receptor types 1, 2, 3, and 4 respectively
HWB: human whole blood
MIA: monoiodoacetate
mPGES‐1 and mPGES‐2: microsomal prostaglandin E synthase 1 and 2
NSAID: non‐steroidal anti‐inflammatory drug
OA: osteoarthritis
RA: rheumatoid arthritis

## Introduction

Chronic inflammation of the synovial joint is a common feature of both osteoarthritis (OA) and rheumatoid arthritis (RA) and is associated with pain, swelling, synovial hyperplasia, destruction of cartilage and bone, leading to reduced mobility (Gardner 1994; Sokolove and Lepus 2013). While the pathophysiology of the disease processes and the mediators involved in OA and RA are not fully understood, cyclooxygenase‐2 derived prostaglandins play a key role in initiating inflammation and perpetuating the signs and symptoms of the diseases by activating multiple inflammatory cells (Smith 1989, Funk 2001; Smyth et al. 2009). Prostaglandins (PGs) represent a family of bioactive lipid mediators that are produced from arachidonic acid via a multistep enzymatic sequence. Although multiple prostaglandins are produced, PGE_2_ is the most prominent prostanoid responsible for the inflammation and pain in both OA and RA. In animal models of arthritis, a neutralizing antibody to PGE_2_ is as effective as nonsteroidal anti‐inflammatory drugs (NSAIDs) treatment (Portanova et al. 1996) in reducing inflammation and hyperalagesia. PGE_2_ synthesis is catalyzed by a specific terminal synthase called microsomal prostaglandin E synthase 1 (mPGES‐1) (Jakobsson et al. 1999). Gene deletion and pharmacological studies indicate that the inhibition of mPGES‐1 is effective in reducing signs and symptoms of arthritis in several animal models (Trebino et al. 2003; Xu et al. 2008; Chandrasekhar et al. 2016). NSAIDs and selective cyclooxygenase inhibitors (coxibs) provide symptomatic relief in human arthritis by blocking the production of PGE_2_ through the inhibition of cyclooxygenase‐1and/or cyclooxygenase‐2 (FitzGerald 2003; Rainsford 2007). While each of these approaches of PGE_2_ modulation provide clinical efficacy, serious side effects have been associated with these drugs since they also interfere with the physiological roles of PGE_2_ in the kidney, gastrointestinal tract, cardiovascular, and immune system (Fitzgerald 2004; FitzGerald and Patrono 2001; Flavahan 2007).

The biological actions of PGE_2_ are mediated through four different membrane bound G‐protein‐coupled receptors (GPCRs), EP1‐4 (Clark et al. 1989; Coleman et al. 1994; Narumiya et al. 1999; Breyer et al. 2001 Sugimoto and Narumiya 2007). PGE_2_ binds with high affinity to each of the structurally distinct gene products. Upon ligand binding, the cytoplasmic C‐terminal regions of the receptors become associated with distinct G‐protein subunits that produce a variety of signal transducers. EP2 and EP4 couple to Gs proteins to stimulate cAMP induction. EP1 couples to Gq proteins leading to an increase in intracellular Ca2 +  accumulation and the EP3 receptor couples to Gi protein and serves to inhibit cAMP (Bos et al. 2004; Narumiya et al. 1999). EP4 is widely expressed in a variety of cells (monocytes/macrophages, platelets, and neuronal cells) and tissues. Gene deletion studies suggest that deletion of the EP4 receptor (but not EP1, EP2, or EP3 deletion) protected against arthritis generated in mice upon type II collagen monoclonal antibody challenge, while wild‐type mice developed inflammatory arthritis and related changes in histology (McCoy et al. 2002).

Since the EP4 receptor is not known to be associated with direct alteration in other prostanoids, there has been considerable interest in developing EP4 antagonists as potential safer anti‐inflammatory molecules. Recently, several EP4 antagonists have been described and efficacy has been demonstrated in a variety of animal models of arthritis (Nakao et al. 2007; Murase et al. 2008; Clark et al. 2008; Xu et al. 2008). CJ‐023,423, a selective EP4 antagonist has been shown to be efficacious in a prospective placebo controlled study for signs and symptoms of OA in dogs (Rausch‐Derra et al. 2016). Recently, we have described several EP4 antagonists that are highly selective and potent (Blanco et al. 2016a,b,c; Schiffler et al. 2015). In this report, we describe the pharmacological characteristics of the molecules and show that they are highly effective in reducing the signs and symptoms in a variety of animal models of analgesia and inflammation.

## Materials and Methods

### EP4 antagonists

Compounds 1, 2, and 3 were prepared as described (Blanco et al. 2016a,b,c, Schiffler et al. 2015). Reference EP4 antagonists CJ‐023,423 and CJ‐042,794 were prepared as described (Nakao et al. 2002; (Yamagishi et al. 2005) respectively. The reference EP1 antagonist MF266‐1, EP2 antagonist, and EP3 antagonist MF266‐3 were prepared as described (Ducharme et al. 2005; Skerratt and Dack 2009; Juteau et al. 2001) respectively.

### EP4 cAMP antagonist assays

Human EP4 (GenBank Accession # AY429109, Clone ID PER0400000, UMR cDNA Resource Center, Rolla, MO) was stably expressed in HEK293 cells. Rat EP4 cDNA (GenBank Accession# NM_03276) was cloned into pcDNA 3.1 vector and stably transfected into HEK293 cells. The cell lines were maintained in DMEM (invitrogen) supplemented with 10% fetal bovine serum (FBS), 1 mmol/L sodium pyruvate, 10 mmol/L HEPES, 500 *μ*g/mL geneticin and 2 mmol/L l‐glutamine. Confluent cultures were grown at 37°C in an atmosphere containing 5% CO_2_. Cells were harvested using 0.25% Trypsin‐ EDTA, suspended in freeze media at a density of 10^7^cel1s/mL, and aliquots were stored in liquid nitrogen. Immediately prior to the assay, cells were thawed with DMEM and suspended in cAMP assay buffer (HBSS with 0.1% BSA, 20 mmol/L HEPES and 200 *μ*mol/L IBMX).

The inhibition of PGE_2_‐stimulated cAMP production by EP4 antagonists was measured using homogeneous time resolved fluorescence technology (HTRF; Cisbio, cat # 62AM4PEB). 4000 cells were incubated with 50 *μ*L cAMP assay buffer containing EC_80_ of PGE_2_ (0.188 nmol/L PGE2, Sigma, cat# P5640) and antagonists at room temperature for 20 min. CJ‐023,423 served as the positive control. To measure the cAMP levels, cAMP‐d2 conjugate and anti cAMP‐cryptate conjugate in lysis buffer were incubated with the treated cells at room temperature for 1 h. The HTRF signal was detected using an EnVision plate reader (Perkin‐Elmer, Inc., Waltham, MA, USA.) to calculate the ratio of fluorescence at 665 to 620 nm. The raw data were converted to cAMP (nmol/L) using a cAMP standard curve generated for each experiment. Data were analyzed using a 4‐parameter nonlinear logistic equation. Results are expressed as the geometric mean ± standard deviation or geometric mean ± standard error as noted.

### EP1, EP2, EP3, and EP4 binding assays

Receptor binding assays were done using membrane preparations of HEK 293 cells transfected with various prostanoid receptors using previously published procedure (Nakao et al. 2007). EP1 and EP4 membranes were prepared from recombinant HEK293 cells stably expressing human EPl receptor (GenBank Accession # AY275470, Clone ID PER0100000, UMR cDNA Resource Center, Rolla, MO) or human EP4 receptor. EP2 and EP3 membranes were prepared from HEK293 cells transiently transfected with human EP2 receptor (GenBank Accession # AY275471, Clone ID PER0200000, UMR cDNA Resource Center, Rolla, MO) or human EP3 receptor (GenBank Accession # AY429108, Clone ID PER3VI0000, UMR cDNA Resource Center, Rolla, MO) plasmids. Frozen cell pellets were homogenized in homogenization buffer (10 mmol/L Tris‐HCl, pH7.4, 250 mmol/L sucrose, 1 mmol/L EDTA, 0.3 mmol/L indomethacin and protease inhibitor cocktail with EDTA (Roche Molecular Biochemicals, catalog # 1691498)) using a Teflon/glass homogenizer. After a low speed centrifugation (200*g* for 15 min at 4°C) the supernatant was subjected to high speed centrifugation (30,000*g* for 60 min at 4°C). Membrane protein preparation was aliquoted and quick frozen on dry ice prior to storage at −80°C. The *K*_d_ values for [^3^H]‐PGE_2_ binding to each receptor were determined by saturation binding studies or homologous competition. Compounds were tested in a 96‐well format using a three‐fold dilution series to generate 10‐point curves. Diluted compound was incubated with 20 *μ*g/well EP1, 10 *μ*g/well EP2, l *μ*g/well EP3 or 10–20 *μ*g/well EP4 membrane for 90 min at 25°C in the presence of 0.3–0.5 nmol/L [^3^H]‐PGE_2_ (PerkinElmer, 118–180 Ci/mmol). The binding reaction was performed in 200 *μ*L MES buffer (10 mmol/L MES, pH 6.0, 10 mmol/L MgCl_2_, and l mmol/L EDTA) using polystyrene 96‐well plates. To determine the effects of EP4 antagonists, membrane preparations were treated with various concentrations of compounds with a constant concentration of (0.3 nmol/L) [^3^H]‐PGE_2_. The membranes were harvested by filtration (TomTek harvester), washed four times with cold buffer (10 mmol/L MES, pH 6.0, 10 mmol/L MgCl_2_), dried in a 60°C oven, and the radioactivity was quantified as counts per min (CPM) using a TopCount detector. Percent specific binding was calculated as the percent of the binding in the absence of any inhibitor, corrected for binding in the presence of 2 *μ*mol/L PGE_2_. Data were analyzed using a 4‐parameter nonlinear logistic equation. *K*_i_ Conversion from IC_50_ Values (*K*_i_ = IC_50_ (1 +  [L]/*K*_d_) where [L] is the ligand concentration). Results are expressed as the geometric mean ± standard deviation or geometric mean ± standard error as noted.

### Off‐target activity

The off‐target activity related to prostaglandin pathway (Table 2) were obtained at CEREP (Blanco et al. 2016c).

### Whole blood assays

The in vitro whole blood assay was conducted using established procedures (Murase et al. 2008). Blood was collected from normal volunteer donors into sodium heparin vacutainer tubes. Donors had not taken glucocorticoids within 2 weeks of the donation. All tubes/donor were pooled into 50 mL Falcon conical centrifuge tubes and 98 *μ*L/well of blood was distributed into 96 well tissue culture plates (Falcon 3072). Compounds were diluted into DMSO to 100 X final and 1 *μ*L/well in triplicate was added to the blood to give 7 point concentration response curves. The blood was pretreated with the compounds at 37°C, 5% CO_2_ in a humidified atmosphere, for 30 min, after which 1 *μ*L/well of a solution of 1 mg/mL of lipopolysaccharide (LPS) (Sigma, serotype 0111:B4) in 0.2 mg/mL‐ bovine serum albumin (BSA)/PBS ± 1 *μ*mol/L PGE_2_ (Cayman 14010) was added to give a final concentration of 10 *μ*g/ml LPS ± 10 nmol/L PGE_2_. The plates were incubated for 20–24 h at 37^°^C, 5% CO_2_, in a humidified atmosphere. The plates were centrifuged at 1800*g*, 10 min at 22°C, in an Eppendorf 5810R centrifuge. Plasma was removed from the cell layer and was transferred to v‐bottom polypropylene plates. TNF*α* ‐levels in plasma were quantitated by a commercially available enzyme immunoassay (R&D Systems, DY210), using Immulon 4 HBX plates (Thermo 3855) and 3, 3′, 5, 5′ tetramethylbiphenyl‐4, 4′‐diamine substrate (KPL 50‐76‐03). The plates were read at A_450_‐A_650_ on a plate reader (Molecular Devices Versamax) using SOFTmaxPRO (v. 4.3.I) software. IC_50_s were calculated using Graphpad Prism (v. 4) nonlinear regression, sigmoidal dose response curve fitting. Results are expressed as the geometric mean ± SD.

### Animal models

Studies were either run at Eli Lilly or at Covance Incorporated, Greenfield, Indiana. All experiments were carried out according to animal care and use protocols approved by the Eli Lilly and Covance Institutional Animal Care and Use Committees.

For the monoiodoacetate (MIA) and adjuvant induced arthritis (AIA) studies, male Lewis rats (Envigo, Indianapolis, IN) of approximately 8 weeks of age at the time of disease induction were used. For collagen induced arthritis studies (CIA) female Lewis rats (Charles River) weighing 150–170 g were used. Rats were housed in groups of 2 or 3 per cage and maintained in a constant temperature, and on a 12 h light/12 h dark cycle. Animals had free access to food and water at all times except during data collection.

### Rat MIA model of pain

The right knees of rats were injected with 0.3 mg of MIA (Sigma Aldrich) in 50 *μ*L of saline and the left knees with 50 *μ*L of saline on day zero. These intra‐articular injections are directly into the synovial cavity of the flexed knee joint through the patella ligament. Twelve days later, the rats were randomized into groups of five rats using body weight and the Block Randomized Allocation Tool. For each study rats were dosed with vehicle (10% Acacia plus 0.05% antifoam) and either compound 1 or compound 2 at doses of 0.3, 1, 3, and 10 mg/kg. Reference compound CJ‐042,794 was also tested in the MIA model on day 14 post MIA injection at doses of 1, 3, 10, 30, and 50 mg/kg with the vehicle being 10% Solutol in PEG400 (*n* = 4 rats per group). The nonsteroidal anti‐inflammatory drug (NSAID) diclofenac was included in all studies at a dose of 5 mg/kg as a positive control. All dosing was once only by oral gavage and dose volume was 5 mL/kg and dosing was staggered by 10 min for each rat. For compound 1 pain was measured 30 min post dose and for compound 2 and CJ‐042,794 pain was measured 1 h post‐dose. These times were chosen as they represented *T*_max_ for each compound. Pain was measured using incapacitance testing. This test measures the difference in hind paw weight bearing between the MIA and saline injected knees, and for these studies each value represents the average of three separate measurements acquired over a one‐second period per rat.

Results from compound 1 will not be reported as they have been previously published (Blanco et al. 2016b).

### AIA model of inflammation

On Day 0, paw widths of both right and left paws were measured between the lateral and dorsal surfaces using calipers, and a mean paw thickness for each rat was calculated by averaging these measurements. Rats were then inoculated intradermally with 0.25 mg of adjuvant (M. Tuberculosis H37 RA from Difco) in 100 *μ*L of mineral oil at the base of the tail to induce adjuvant disease. Eleven days post inoculation paw widths of both right and left paws were measured again, and the percent change from the Day 0 reading was calculated. This measurement, along with the body weight of the rats, was used to randomize the animals into five groups of eight animals. For each study rats were dosed with vehicle (10% Acacia plus 0.05% antifoam) and either compound 1 or compound 2 at doses of 1, 3, and 10 mg/kg. Reference compound CJ‐023,423 was also tested in the AIA model at doses of 30, 60, and 100 mg/kg. The nonsteroidal anti‐inflammatory drug (NSAID) diclofenac was included in all studies at a dose of 10 mg/kg as a positive control. Dosing was once daily (twice daily for CJ‐023,423) by oral gavage for 4 days at a dose volume of 5 mL/kg. On the fifth day after dosing started (day 15 post adjuvant injection) the rats were euthanized without further dosing and paw width measured again. The percent increase in mean paw thickness from day 11 to day 15 was then calculated as a measure of paw swelling which is indicative of the amount of inflammation in the paws measured. Results from compound 1 will not be reported as they have been previously published (Blanco et al. 2016b).

### Type‐II CIA model of inflammation and autoimmunity

On day 1 of the study ankle widths are measured across the joint (left side to right side) at the thickest point with calipers and rats immunized intradermally near the base of the tail with 0.4 mL of 1 mg/mL type II collagen (Elastin Products) emulsified with incomplete Freund's adjuvant (Sigma Aldrich). A second booster injection booster of 0.4 mL of the collagen emulsion was given on day 8 of the study. Ankle widths were measured on day 8 before the collagen injection and every 2–3 days thereafter until study end. On day 11 rats were randomized into five groups of eight animals based on inflammation (redness and/or swelling) in their hind paws and body weight. Rats were then dosed with vehicle (10% Acacia plus 0.05% antifoam) and compound 3 at doses of 3, 10, and 30 mg/kg. Prednisolone was included at a dose of 10 mg/kg as a positive control. Dosing was by oral gavage once daily for seventeen days at a dose volume of 5 mL/kg. On day 28 rats were euthanized and hind paws and knee joints collected for histology. Ankles and knees were fixed in neutral buffered formalin, decalcified, embedded in paraffin, and sectioned according to standard histological methodology and the joints were then scored by a pathologist using published scoring criteria (Bendele 2001; Levine et al. 2014).

### Statistical analysis

Data are presented as means with standard error of the means (SEM). Data were evaluated by one way analysis of variance (ANOVA). Differences between groups were considered to be significant if the *P* < 0.05.

## Results

### Identification of novel human EP4 receptor antagonists

**Figure 1:**
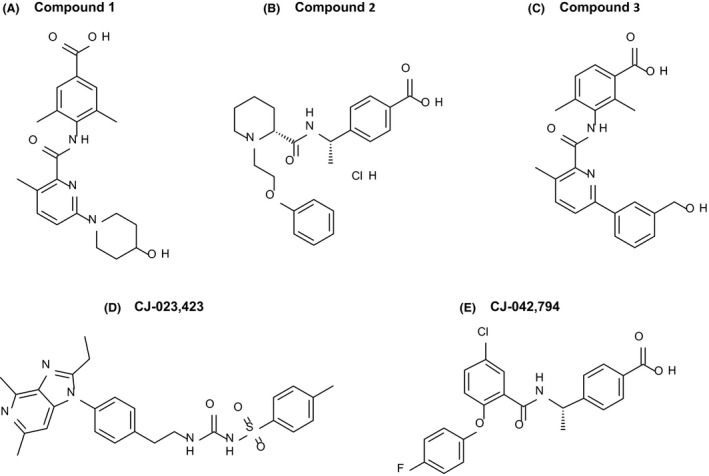
Chemical structure *of EP4* antagonists. Compounds 1, 2, and 3 are the newly described EP4 antagonists. CJ‐023,423 and CJ‐042,794 are reference EP4 antagonists.

**Figure 2:**
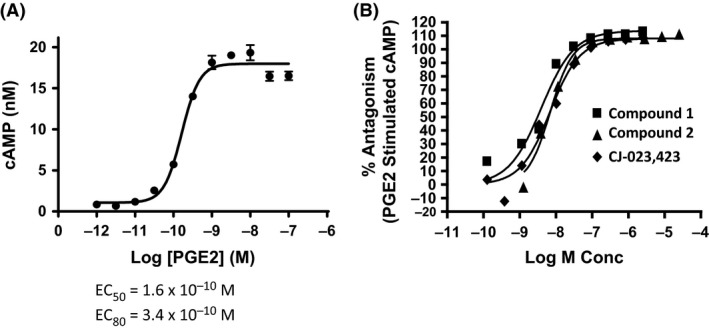
*EP4* antagonists block *cAMP* production in *HEK‐EP4 Cells*. HEK‐293 cells stably transfected with human EP4 cDNA were treated with various concentrations of PGE_2_ (Panel A) for 20 min at room temperature and cAMP levels were determined as described in Materials and Methods. To determine the effects of EP4 antagonists, cells were treated with various concentrations of compounds followed by PGE_2_ at EC_80_ concentration for 20 min and the cAMP levels were determined. The results are expressed as % inhibition in comparison to cAMP levels obtained at EC_80_ concentration of PGE_2_ (Panel B). The figure is a representative of results from experiments of *n* = 8 for compound‐1, *n* = 10 for compound‐2 and *n* = 6 for CJ‐023,423.

The structures of three optimized EP4 antagonists (Compound 1, Compound 2, and Compound 3) representing different scaffolds, and two reference EP4 inhibitors CJ‐023,423 and CJ‐042,794 are shown in Figure 1. EP4 antagonists were initially identified based on the ability of compounds to antagonize PGE_2_ stimulated cAMP production by HEK293/EP4 cell line. HEK 293 cells stably transfected with human EP4 cDNA were evaluated for their ability to produce cAMP in response to various concentrations of PGE_2_. The results (Fig. 2A) show a robust concentration‐dependent response to PGE_2_ with an EC_50_ of 0.16 nmol/L and an EC_80_ 0.34 nmol/L. To demonstrate EP4 antagonism, freshly thawed cells were treated first with various concentrations of compounds followed by PGE_2_ at EC_80_ concentration. Then the cells were assayed for cAMP production. A representative example of the antagonist activity of the compounds is shown in Figure 2B. Both compounds 1 and 2 completely blocked the PGE_2_‐generated cAMP in a concentration‐dependent manner with IC_50_ values of about 6 nmol/L. The activities were comparable to a reference EP4 antagonist, CJ‐023,423 (IC_50 _= 12 nmol/L). The IC_50_ of compound 3 was 2.4 nmol/L in a separate assay. All compounds also blocked cAMP production in rat EP4 transfected HEK293 cells with IC_50_ values comparable to the values against human EP4 receptor (Table 1).

**Table 1:** Summary of in vitro activities

Assay	Compound‐1	Compound‐2	Compound‐3	CJ‐023,423	CJ‐042,794
cAMP Antagonism *(hEP4)*	IC50 (nmol/L) ± SEM	IC50 (nmol/L) ± SEM	IC50 (nmol/L) ± SEM	IC50 (nmol/L) ± SEM	IC50 (nmol/L) ± SEM
Human EP4	5.6 ± 1.1; *n *= 8	5.6 ± 1.0; *n * = 10	2.4 ± 1.4; *n* =5	11.7 ± 3.0; *n* = 6	4.5 ± 1.85; *n* = 153
Rat EP4	12.3, *n* = 1	15.4; *n* = 1	1.0, *n* = 1	13.1 ± 1.0; *n* = 5	20.6 ± 1.8; *n* = 20
hEP1‐4 receptor binding	Ki (nmol/L) ± SEM				
EP1	>17500; *n* = 2	>17500; *n* = 3	>17500; *n* = 1	>22900; *n* = 1	>26700; *n* = 2
EP2	>18900; *n* = 4	1210 ± 509; *n* = 6	>18900; *n* = 1	>21600; *n* = 2	931 ± 457; *n* = 6
EP3	>14000; *n* = 4	>14000; *n* = 5	>14000; *n* = 1	>19500; *n* = 1	>15500; *n* = 1
EP4	58 ± 12; *n* = 10	41 ± 7; *n* = 12	2.07 ± 1.31; *n* = 8	449 ± 123; *n* = 8	12.6 ± 7.36; *n* = 6
Human whole blood	IC50 (nmol/L) ± SD	IC50 (nmol/L) ± SD	IC50 (nmol/L) ± SD	IC50 (nmol/L) ± SD	IC50 (nmol/L) ± SD
(Reversal of PGE2 Inhibited TNFα)	123 ± 80; *n* =8	123 ± 88; *n* =12	42 ± 17; *n* = 9	1560 ± 105; *n* = 79	840 ± 630; *n* = 15

Data are expressed as geometric mean ± standard deviation or standard error as noted.

### Selectivity of EP4 receptor antagonists

**Figure 3:**
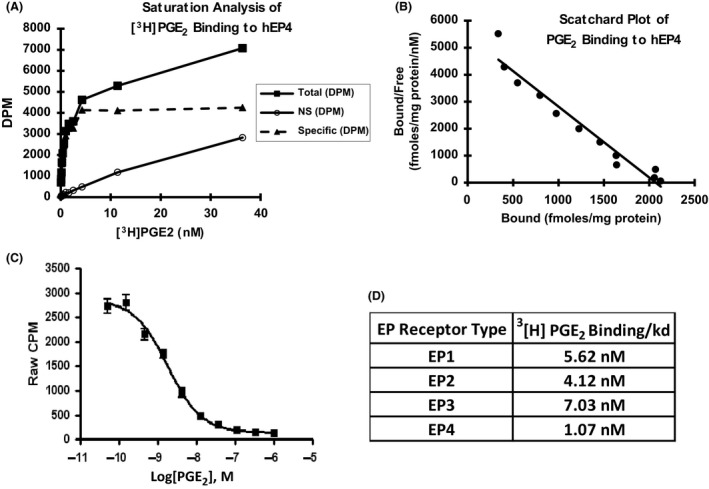
Characterization of EP receptor binding. Membrane preparations from HEK 293 cells transfected either stably (EP1 cDNA or EP4 cDNA) or transiently (EP2 cDNA or EP3 cDNA) were treated with ^3^[H]PGE_2_ (0.3–0.5 pM) for 90 min and the radioactive binding was determined (refer to materials and methods). The specific binding was determined by subtracting binding to membrane preparations from HEK 293 cells that contained no EP receptors (Panel A). To further characterize EP4 receptor, HEK293/EP4 cell membrane preparations were treated with various concentrations of unlabeled PGE_2_ in the presence of constant ^3^[H]PGE_2_ and the radioactive binding determined (Panel C). A scatchard analysis of the binding was done as described in materials and methods.(Panel B). Similar studies were conducted with other EP receptors and the results expressed in Panel D.

The selectivity of the compounds for EP4 receptor was established by comparing the ability of compounds to block PGE_2_ binding to various EP receptors (EP1, EP2, EP3, EP4). Membranes were prepared from HEK293 cell lines that express various EP receptors (see materials and methods). Initially, optimal saturation conditions were established for all receptors using various concentrations of ^3^[H] PGE_2_. The results shown for EP4 binding indicate the binding was linear and saturable, with minimal non‐specific binding (Figs. 3A and B). We next established homologous competition assay for EP1, EP2, EP3, and EP4 receptors using various concentrations of unlabeled PGE_2_ and a constant concentration (0.3–0.5 nmol/L) of ^3^[H] PGE_2_. The results (Fig. 3D) indicate a close range of Kds for all EP receptors in the over‐expressed cell lines. A representative competition result (Fig. 3C) is shown for EP4 indicating a full inhibition of binding.

**Figure 4:**
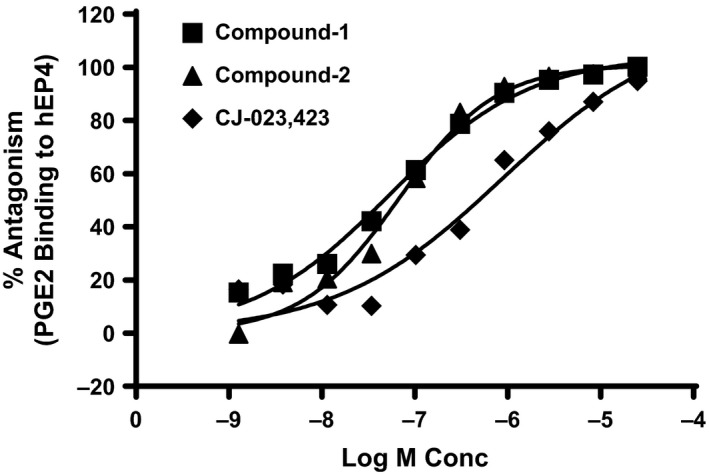
EP4 antagonists block PGE_2_ Binding to EP4. HEK293/EP4 membrane preparations were treated with a constant concentration (0.3 nmol/L) of [^3^H]‐PGE_2_ and various concentrations of indicated compounds for 90 min at room temperature. The results are expressed as % antagonism with membrane preparations that contained no antagonists serving as control (100%). The figure is a representative of results from experiments of *n* = 10 for compound‐1, *n* = 12 for compound‐2 and *n* = 8 for CJ‐023,423.

We next evaluated the ability of Compounds 1, Compound 2 and the reference compound to block ^3^[H]PGE_2_ binding to EP4 receptor. The results shown in Figure 4, demonstrate that the compounds are able to fully antagonize ^3^[H] PGE_2_ binding. The results of the competitive binding assays using EP receptors are summarized in Table 1. They demonstrate that compounds 1 (Ki = 58 nmol/L), 2 (Ki = 41 nmol/L) are 7–10 times more potent than the reference EP4 inhibitor, CJ‐023,423 (Ki = 449 nmol/L) and that Compound 3 was even more potent with a Ki of 2.05 nmol/L. The results further demonstrate that all the three compounds selectively antagonize PGE2 binding to the EP4 receptor. Compound 2 exhibits a 30 fold weaker affinity for EP2 receptor (*K*_i_ = 1.2 *μ*mol/L).

### EP4 antagonists block TNF‐*α* production in human whole blood

**Figure 5:**
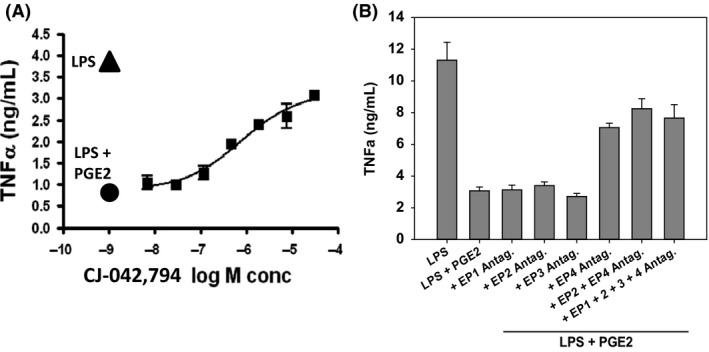
Characterization of *EP4* antagonists on *PGE*_*2*_ inhibition of TNF*α* production in human whole blood in vitro. The pooled whole blood obtained from normal volunteers was pretreated in triplicate with various concentrations of the reference EP4 antagonist (CJ‐042,794) (Panel A) or 10 *μ*mol/L of EP (1, 2, 3 and 4) antagonists (Panel B) at 37°C, 5% CO_2_ in a humidified atmosphere, for 30 min, followed by treatment with a final concentration of 10 *μ*g/mL LPS ± 10 nmol/L PGE_2_ for 24 h 37°C, 5% CO_2_ in a humidified atmosphere. The plasma was collected by low speed centrifugation of microtiter plates and assayed for TNF‐*α* levels. Panel A: ▴ LPS control; ●LPS+PGE_2_; ■ compound + LPS + PGE_2_; Panel B: TNF*α* values in the presence of EP (1, 2, 3 and 4) antagonists.

**Figure 6:**
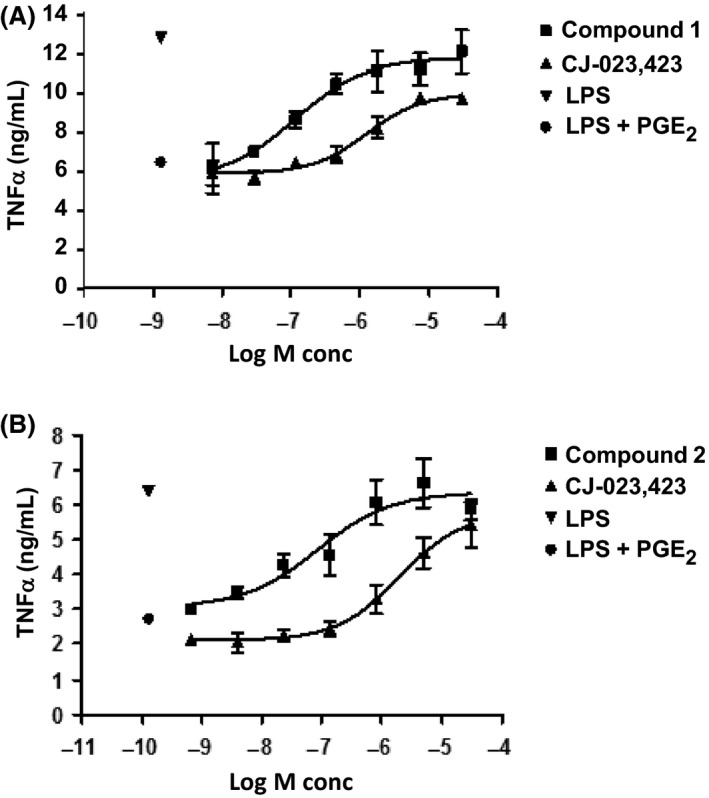
Concentration‐dependent Reversal of PGE_2_ effects on TNF*α* production by EP4 antagonists Pooled human whole blood samples (triplicate) from normal volunteers were pre‐treated with various concentrations of compound 1(Panel A), compound 2(Panel B) or reference EP4 inhibitor (CJ‐023,423) for 30 min and TNF*α* production determined as described in Materials and Methods. The figure is a representative of results from experiments of *n* = 8 for compound‐1, *n* = 12 for compound‐2 and *n* = 79 for CJ‐023,423. Panel A and B: ▾LPS control;● LPS+PGE_2_;■ compound + LPS + PGE_2_; ▴ CJ‐023,423 + LPS + PGE_2._

Previous studies have demonstrated that macrophages express EP4 receptors and that PGE_2_ inhibited LPS stimulated TNF‐*α* production, which was abolished in EP4‐deficient macrophages (Meha et al. 1997; Nataraj et al. 2001). In addition, EP4 antagonists have been shown to block PGE_2_ inhibition of TNF‐*α* in isolated monocytes as well as in whole blood cells (Murase et al. 2008). In order to further validate the activity of EP4 antagonists in a clinically relevant matrix (human whole blood), we evaluated whether compounds 1 and 2 were effective in reversing PGE_2_ suppression of TNF‐*α* in the human whole blood assay. Initially, we established optimal conditions demonstrating that LPS stimulated TNF‐*α* production was suppressed by exogenously added PGE_2_ and demonstrated that the inhibition was reversed by the reference EP4 antagonist (CJ‐042,794, Fig. 5 A). The results also show that only EP4 antagonist and not other EP receptor antagonists tested (EP1, EP2, and EP3) were effective in blocking PGE_2_ effects on TNF‐*α*.production (Fig. 5 B). We next examined the effects of compounds 1 and 2 in the whole blood assay. The results shown in Figure 6 demonstrate that both the compounds blocked PGE_2_ effects on TNF‐*α* production in human whole blood. The IC_50_ values for both compound was 123 nmol/L, and the value for compound 3 was 42 nmol/L (Table 1). The values for the two reference EP4 inhibitors (CJ‐023,423 and CJ‐042,794) were 1560 nmol/L and 840 nmol/L respectively (Table 1).

### EP4 antagonists are efficacious in a rat MIA model of pain

**Figure 7:**
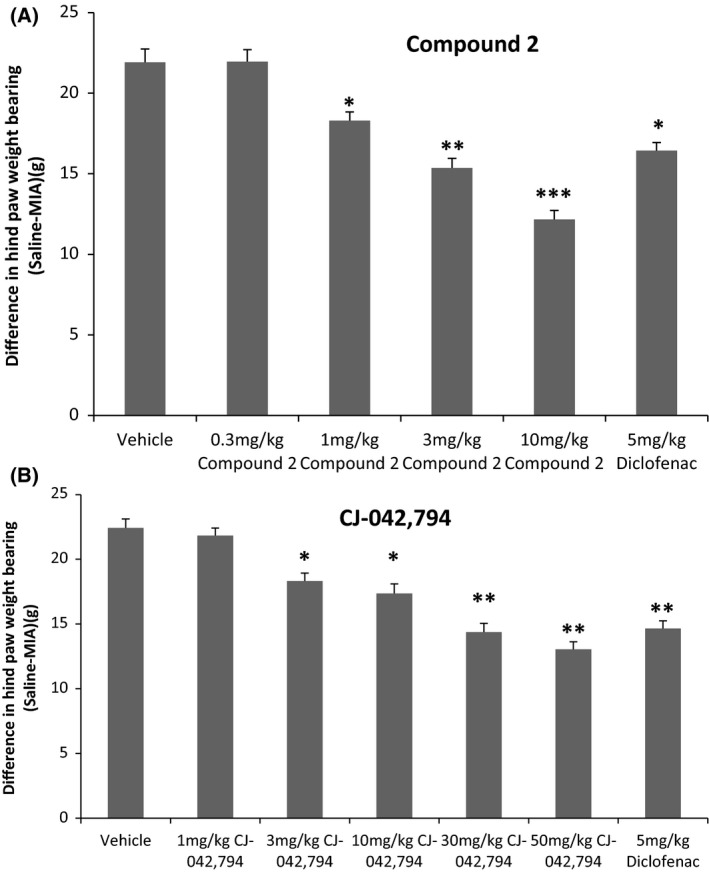
Assessment of pain efficacy in rat MIA model. Rats injected with MIA was treated with various doses of EP4 antagonists (compound 2 or CJ‐042,794) or the NSAID diclofenac (5 mg/kg) and pain was measured 1 h post dosing by an incapacitance test (materials and methods). The data are presented as mean ± SEM where group size *n* is 4 or 5. Statistical comparison to vehicle: Dunnett's test and between groups Tukey HSD (*/**/***P <* *0.05).

We next evaluated the ability of EP4 inhibitors to block the pain resulting from joint injury caused by the intra‐articular injection of monoiodoacetate. Previous studies have established that the injection of monoiodoacetic acid (MIA) into the knee joint of rats produces an acute inflammatory insult, joint degeneration, and pain (Bove et al. 2003). The pain resulting from the joint injury can be measured via differential weight bearing of the hind legs using an incapacitance tester (Benschop et al., 2014). In order to evaluate the analgesic efficacy, rats were injected with 0.3 mg of MIA on Day 0 and 12 days later were dosed with vehicle, EP4 inhibitors at the indicated doses, or 5 mg/kg of the NSAID diclofenac. The pain was measured using incapacitance testing 30 min (compound 1) or 1 h (compound 2) post dosing. Efficacy was measured by the ability of a compound to partially normalize weight distribution. The results for compound 2 are shown in Figure 7A (compound 1 results have been published previously). Compound 2 was effective at 1, 3, and 10 mg/kg (*P *<* *0.05 by Dunnett's test). with each increasing dose of compound 2 being significantly different from the preceding dose (*P *<* *0.05 by Tukey HSD). Similar results were also achieved with reference EP4 antagonist CJ‐042,794 (Fig. 7B) with the 3, 10, 30, and 50 mg/kg doses being significantly different from vehicle (*P *<* *0.05 by Dunnett's test) and the 30 and 50 mg/kg doses being significantly different from the 1, 3, and 10 doses (*P *<* *0.05 by Tukey HSD).

### Inhibition of Inflammatory response in rat adjuvant AIA by EP4 antagonists

**Figure 8:**
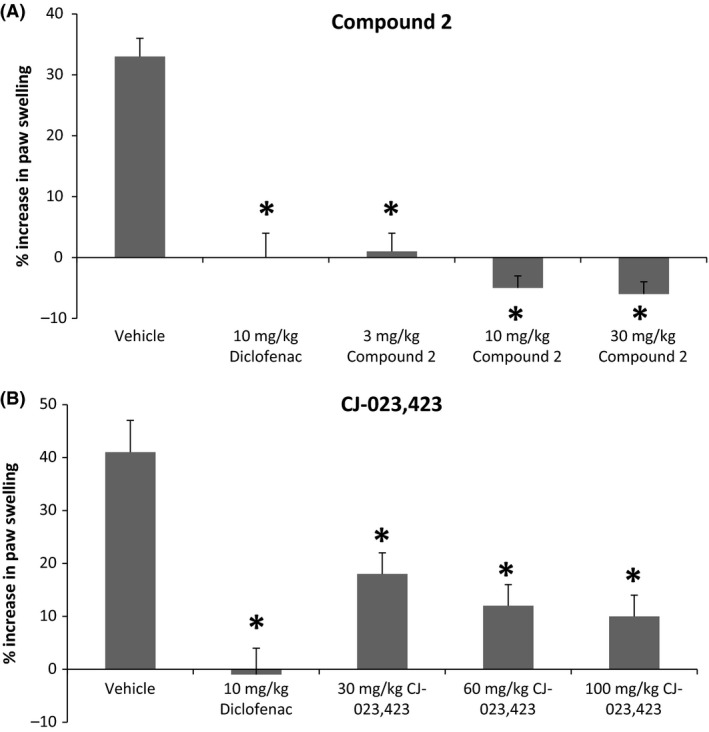
Efficacy in adjuvant induced arthritis model of inflammation. Rats injected with adjuvant for 11 days were treated with various doses of EP4 antagonists (compound 2 or CJ‐023,423) diclofenac, or vehicle for 4 days and the paw width was determined before and after compound treatments. The data are expressed as % increase in paw swelling. The data are presented as mean ± SEM (*n* = 8). Statistical comparison to vehicle: Dunnett's test (*P <* *0.05).

In order to assess the potential anti‐inflammatory effects of EP4 antagonists, the compounds were evaluated in rats injected with complete Freund's adjuvant induced arthritis (AIA) model as described in the methods section. The percent increases in mean paw thickness from Day 11 to Day 15 were calculated as a measure of paw swelling, indicative of the amount of inflammation in the paws measured. Compound‐2 significantly inhibited increases in paw inflammation (swelling) compared to vehicle at all 3 doses tested, as did diclofenac at l0 mg/kg (Fig. 8A; *P *<* *0.05 by Dunnett's test). Results for compound 1 are not shown as they have been published previously (Blanco et al. 2016b). Similar results were also achieved with reference EP4 antagonist CJ‐023,423 (Fig. 8B).

### EP4 antagonists are effective in Rat CIA model

**Figure 9:**
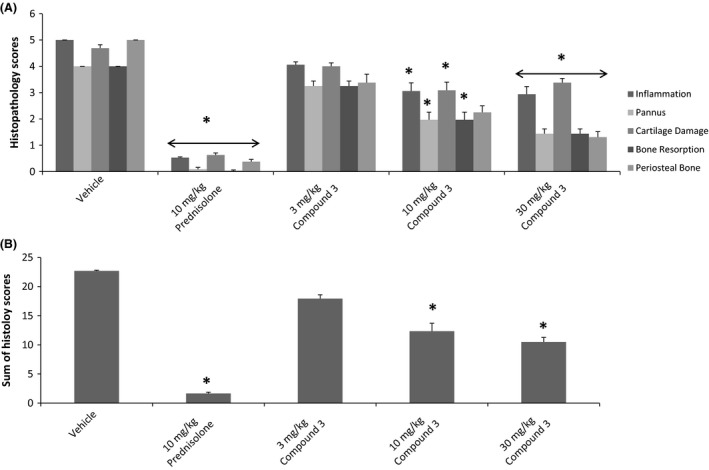
EP4 Antagonists in Collagen II induced arthritis model. Arthritis was induced in rats by intradermal injection of type II collagen as described (Materials and Methods). Treatment with indicated doses of EP4 antagonist (compound 3) or prednisone (10 mg/kg) started on day 11 post collagen injection. After 17 days of treatments, hind paws and knee joints were collected for histology, ankles and knees were assessed for histopathology. Panel A: Individual scores; Panel B: Total histopathological scores; Panel C: Periosteal Bone measure.

**Figure 10:**
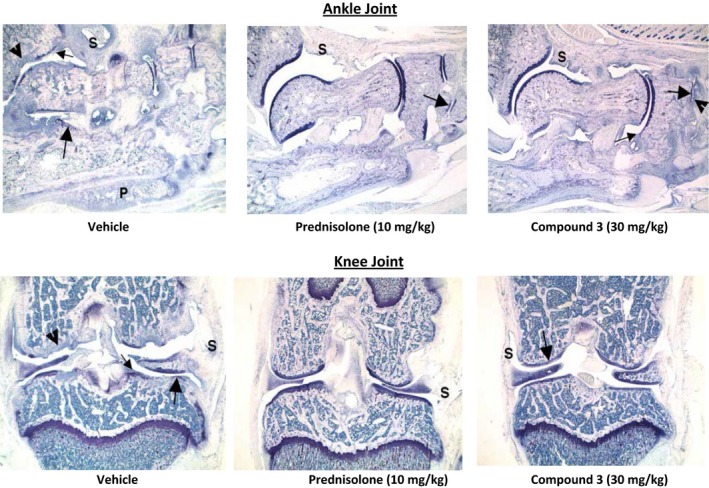
Representative Photomicrographs of Ankle (Top) and Knee (Bottom) joints. Top Panel: Ankle from vehicle control has severe inflammation (s) and cartilage damage (large arrow) with marked pannus (small arrow), bone resorption (arrowhead) and periosteal bone formation (P). Ankle from arthritic animal treated 10 mg/kg prednisolone has minimal inflammation (S) and minimal cartilage damage (large arrow); Ankle from compound 3 treated rats has moderate inflammation (S) and cartilage damage (large arrow), mild pannus (small arrow) and mild periosteal bone formation (P). Bottom Panel: Knee form vehicle control has severe inflammation (S) and cartilage damage (large arrow) with marked pannus (small arrow), bone resorption (arrowhead) and bone resorption (P). Knee from arthritic animal treated 10 mg/kg prednisolone has very minimal inflammation (S) and cartilage damage not visible at this magnification (large arrow); Knee from compound 3 treated rats has minimal inflammation (S) and cartilage damage (large arrow), very minimal pannus (small arrow) and bone formation (P) not visible at this magnification.

Previous studies have shown that PGE_2_ plays a key role in inflammatory pathway in animal models of autoimmune disease and that the EP4 receptor is critical in mediating the pro‐inflammatory and immune‐modulatory activity leading to the development of arthritis in these models (Smith 1989, Funk 2001; Smyth et al. 2009). We therefore evaluated whether the EP4 antagonists were effective in blocking the disease phenotype in a rat model of type II collagen induced arthritis. Initial evaluation of joints was done by the assessment of ankle thickness which showed significant reduction with all three doses of compound 3 and prednisolone (data not shown). The ankle and knee joints were evaluated histologically for various signs of inflammation and joint destruction (inflammation, pannus, cartilage destruction, and periosteal bone formation) as described before (Levine et al. 2014). As can be seen from figure 9, ankles from animals with CIA and treated with vehicle had overt inflammation, cartilage damage, pannus formation, bone resorption, and periosteal bone formation (Fig. 9). Similar results were seen for the knee joints of vehicle treated animals (data not shown), Animals treated with 10 mg/kg of prednisolone had significant (87–99%) reductions in all ankle scores and a 93% reduction in summed scores (Fig. 9). Similar scores were seen for the knee joint (data not shown). Ankles from animals treated with compound 3 had significant reductions in nearly all parameters and in summed scores at 10 and 30 mg/kg (Fig. 9) Representative photomicrographs of the ankle and knee joints from vehicle, prednisolone, and compound 3 are shown in Figure 10.

## Discussion

We report here the identification and pharmacological characterization of novel EP4 antagonists. The molecules representing different chemical scaffolds are highly potent, selective and are effective in (1) blocking PGE_2_ stimulated cAMP production, (2) radio‐labeled PGE_2_ binding to EP4 receptors, and in (3) reversing PGE_2_ mediated modulation of TNF‐α production in human whole blood culture. Furthermore, the molecules were effective in alleviating pain and inflammation associated with rat MIA and AIA models respectively. Finally, an EP4 antagonist also significantly reduced the development of inflammation and tissue destruction in a rat model of collagen type II arthritis.

PGE_2_ plays a central role in eliciting inflammation associated with arthritic conditions (Ricciotti and Fitzgerald 2011). NSAIDs and coxibs which block PGE_2_ have been the mainstay in the treatment of the signs and symptoms of arthritis, but have significant safety concerns related to gastro‐intestinal bleeding as well as cardiovascular adverse effects because of the blockage of all prostanoids that play critical roles in normal physiological functions. Therefore, there is a continued interest in developing safer alternatives. Two key approaches have involved by either developing microsomal prostaglandin E synthase inhibitors that selectively block PGE_2_ or targeting the key PGE_2_ receptor that is critical in inflammation. In this report, we describe novel agents that block the action of EP4, a PGE_2_ receptor, which have been implicated as a key driver of inflammation and pain (McCoy et al. 2002).

PGE_2_ actions are mediated through four distinct G‐protein coupled receptors which are differently distributed in tissues (Coleman et al. 1994;Narumiya et al. 1999; Breyer et al. 2001). The receptors transmit signals via specific G‐proteins that result in the production of distinct mediators (Bos et al. 2004; Narumiya et al. 1999). The EP4 receptor primarily couples to Gs protein resulting in cAMP production. Several lines of evidence suggest that EP4 plays a central role in PGE_2_ mediated inflammation, pain, and tissue destruction associated with OA and RA. EP4 receptor has been shown to be abundantly expressed in the synovium of a rat adjuvant arthritis model (Kurihara et al. 2001) and in the articular cartilage of human osteoarthritis (Li et al. 2009). Deletion of EP4 but not EP1, EP2, or EP3 resulted in protection against the development of arthritic lesions in a murine autoimmune model of arthritis induced by type II collagen (McCoy et al. 2002). Furthermore, an EP4 antagonist was effective in an auto‐immune model of arthritis by blocking Th1 differentiation and Th 17 expansion (Chen et al. 2010). Selective EP4 receptor antagonists have been shown to be effective in various models of arthritis and pain while EP1 or EP3 antagonists were ineffective (Murase et al. 2008; Nakao et al., 2007, Xu et al. 2008; Chen et al. 2010). More recently, EP4 receptor antagonists have been shown to be effective as an analgesic agent in dogs (Rausch‐Derra et al. 2016). Thus, EP4 receptor antagonists are likely to be effective as anti‐inflammatory and analgesic agents for arthritic patients.

The initial identification of EP4 antagonists was facilitated by a functional assay in which HEK293 cells transfected with human EP4 cDNA produced cAMP upon stimulation with PGE_2_. EP4 antagonists were effective in blocking cAMP production and demonstrated high potency against EP4 (IC _50_ of 2–6 nmol/L). The compounds were also active against rat EP4. Compound 3 exhibited slightly greater potency against both human and rat EP4 receptors in comparison to compounds 1, 2 as well as reference EP4 inhibitors.

The selectivity of the compounds was also established by receptor binding assays using membrane preparations of HEK293 cells transfected with various receptors. The receptors exhibited saturable and reversible binding (Fig. 2). The Ki values for all receptors were comparable under the conditions tested. Compounds demonstrated higher potency for EP4 (*K*_i_ of 2–40 nmol/L) in comparison to CJ‐023,423 (Ki of 449 nmol/L) and exhibited high selectivity for EP4. Only compound 2 showed activity (approximately 20 fold weaker) for EP2 (Table 1). Compounds showed no discernible activity against several other prostanoid receptors as well as cyclo‐oxygenases 1 and 2 (Table 2).

**Table 2:** In vitro pharmacology for off‐target activities

Compound	Target	Conc, *μ*mol/L	% Inh
Compound 1	COX2 (h)	10	−10
	COXl (h)	10	−216.00^a^
	TP (TXA2/PGH2) (h); antagonist effect	10	−12.00
	TP (TXA2/PGH2) (h); agonist effect	10	−4.00
	IP (PGI2) (h), agonist radioligand	10	−4.00
	FP(h); agonist radioligand	10	−2.00
	DP1(h); agonist radioligand	10	0.00
Compound 2	COX2 (h)	10	−17
	COXl(h)	10	−24.00
	TP (TXA2/PGH2) (h); antagonist effect	10	−12.00
	TP (TXA2/PGH2) (h); agonist effect	10	−2.00
	IP (PGI2) (h), agonist radioligand	10	9.00
	FP(h); agonist radioligand	10	−9.00
	DP1(h); agonist radioligand	10	−4.00

a Non‐specific assay interference.

The activity of EP4 antagonists were also further established using the whole blood assay which provided a more relevant biological matrix (Murase et al. 2008).This assay is based on the following: (1) LPS stimulation of TNF‐*α* in whole blood is blocked by PGE_2_ and (2) PGE_2_ activity is mediated by EP4 as only reference EP4 antagonist, but not other EP receptor (EP1, EP2, EP3) antagonists was effective (Fig. 5). Compounds 1 and 2 demonstrated a dose‐dependent antagonism of PGE_2_ mediated TNF‐*α* production with full efficacy (Fig. 6) and were 5–10 times more potent than reference EP4 antagonists (Table 1). Demonstration of activity in a biologically relevant matrix would be helpful to determine biochemical efficacy and to serve as potential biomarker assay to facilitate clinical dose determination.

The analgesic and anti‐inflammatory activities of EP4 antagonists were demonstrated using well‐established rat models that are known to be PGE_2_ mediated. Coxibs, NSAIDS, and EP4 antagonists have been shown to be effective in reducing rat model of MIA induced pain as well as in adjuvant induced inflammation and arthritic lesions (Burch et al, 2008). Compounds 1 and 2 were effective in both models with compound 2 exhibiting a slightly more efficacy. Type II Collagen induced arthritis model is an auto‐immune model of arthritis in which EP4 receptors have been shown to be critical in the development of polyarthritis (Chen et al. 2010). Furthermore, EP4 receptors have shown to be involved in PGE_2_ stimulation of Th1 differentiation and Th17 expansion contributing to arthritis and EP4 antagonists were effective in blocking the development of arthritis in a mouse model of type II collagen arthritis (Chen et al. 2010). The EP4 antagonist (compound 3) was highly effective in reducing arthritic lesions in a rat model of type II collagen arthritis as demonstrated by the reduction in quantitative arthritic score for knee joints (Fig. 9) as well as in histopathology of joint (Fig. 10). Collectively these results demonstrate that the EP4 antagonists described here show analgesic, and anti‐inflammatory properties and may be efficacious in treating humans suffering from OA and RA.

In conclusion, we have identified novel EP4 antagonists that are highly selective and potent, and are effective as analgesic and anti‐inflammatory molecules. Traditional NSAIDS and coxibs, while effective, exhibit significant liability because of a variety of side‐effects. EP4 antagonists would be expected to be a safer alternative since it is unlikely to have a direct effect on other prostanoid levels. However, this is speculative and needs to be demonstrated in the clinic. The availability of highly selective and potent molecules should facilitate this possibility.

## Author Contribution

*Participated in Research Design*: Srinivasan Chandrasekhar, Xiao‐Peng Yu, Anita K Harvey, Mark G. Chambers, Matthew J. Fisher. *Conducted Experiments*: Anita K. Harvey, Xiao‐Peng Yu, Xushan Wang, Jennifer L. Oskins, Chaohua Lin, Alison M. Bendele. *Contributed new reagents:* Xiao‐Peng Yu, Anita Harvey, Maria‐Jesus Blanco, Tatiana Vetman, Steven L. Kuklish, Jeremy S. York, Matthew A. Schiffler, Matthew J. Fisher, Alan M Warshawsky. *Performed data Analysis*: Anita K. Harvey, Xiao‐Peng Yu, Xushan Wang, Srinivasan Chandrasekhar, Mark G. Chambers, Alison M. Bendele. *Wrote or contributed to the writing*: Srinivasan Chandrasekhar, Anita K Harvey, Xiao‐Peng Yu, Mark Chambers. [Correction added on 30 May 2017, after first online publication: The listings of “Conducted Experiments” and “Contributed new reagents” within Author Contribution have been edited to reflect the correct contributors for this paper.]

## Disclosure

None declared.
